# Case of Obstipation, with Stercoraceous Vomiting, Etc.

**Published:** 1851-02

**Authors:** 


ART. II.—*Case of Obstipation, with Stercoraceous Vomiting, etc.* Reported by the Editor.

On the 30th of Nov., 1850, at 5, a. m., I was requested to visit Mrs. N., aged about 25, a lady with whom I had had no previous acquaintance. I

found the symptoms and history as follows:—In the early part of the night she had began to suffer from pain in the abdomen, back, and lower portion of the chest, which progressively increased in severity, with occasional remissions, until it was deemed proper to call in medical aid. She had been engaged the day before, and for several days previously, very constantly in *quilting.* I ascertained that she possessed a highly impressible constitution, that her health had been for some time delicate, that she had suffered for a long period from leucorrhea and menorrhagia, and, recently, from diarrhœa. The spine was highly sensitive to pressure. The pulse was not accelerated; skin cool. Friction over the situation of the pain was attended by some relief. When the suffering was severe she threw herself wildly about the bed, and exhibited hysterical symptoms. Bowels had not been moved since the day but one prior to the attack.

Regarding, from the above assemblage of facts, the pain as neuralgic in its character, I prescribed morphia, and sinapisms, and left her at 7, A. M., partially relieved. Slight vomiting occurred during this first visit.

At 9, a. m., finding that the pain persisted, and was still severe, I left directions to continue the morphia in doses of gr. 1-4, and was detained by other engagements until noon. In the mean time the pains continued with such severity that Dr. Mixer, of this city, was called in. Some uterine flowing, in the mean while, occurred. Dr. Mixer prescribed morphia in combination with the acetate of lead. The pain persisted, but was in some measure alleviated, until evening, when she became quite easy, and passed a comfortable night.

On the following morning, Dec. 1, I found her free from pain, pulse but little accelerated, complaining somewhat of nausea, which was attributed to the morphia. Rest and simple nourishment alone were advised, and I did not propose to visit her again until the next day.

At noon I was summoned, and found the pain had returned with great severity, and it was now attended by marked hysterical symptoms—violent sobbing and weeping. She had felt so well during the forenoon that she had received some visits, and became excited. It seemed fair to attribute the renewed attack, in part, to this as the cause. Morphia and hot fomentations were directed. The pain, as before, was not confined to one situation, but shifted from one point to another—now in the abdomen, now in the inferior part of the chest, and frequently in the back. The menorrhagia had ceased the day before, and she had now only the usual menstrual discharge.

The pain continued, with great acuteness, during the evening and night. She took during the night four grains of the sulphate of morphia, and, *per enema,* a drachm of *Tinct. opii.* She occasionally vomited, but this was not a prominent symptom, and did not occur save when fluids were taken into the stomach. By withholding drinks vomiting was prevented. She complained of thirst, and entreated for water. The pulse had now become moderately accelerated—about 100. Tongue moist and slightly furred. No dejection from the bowels since the attack.

On the 2d Dec., the pain continaed, but with less severity. It was occasionally severe, but much of the time not very distressing. During the forenoon of this day vomiting became a prominent symptom, and she began to eject a yellowish, and brownish yellow fluid, very fœtid, having distinctly a stercoraceous odor. The quantity vomited was very copious, and occasionally it contained small portions of stercoraceous substance. The vomiting at first afforded relief, and was encouraged by the patient under the impression that matter so offensive should be expelled. The vomiting of this matter continued through the day. Several quarts were ejected. No dejection occurred from the bowels. The abdomen was slightly tympanitic. Percussion elicited flatness over the right iliac region, but no distension existed in this region. The abdomen was universally tender on pressure, but not extremely so. Friction was well borne, and afforded relief. She was at evening much prostrated. The pulse became frequent, numbering 130, small and feeble. Skin warm. Pain was diminished.

Dr. White saw her in consultation on this evening, and we concurred in directing an enema of T. opii, ℨij, and anodynes, *per orem,* if retained, sufficiently to relieve the pain.

Nov. 3d. She had passed a more comfortable night. She had experienced considerable pain, with restlessness, and vigilance, but less suffering than hitherto. Pulse 120, tolerably developed. The vomiting continued, but the matter vomited was less offensive. It had now a greenish tinge. No dejection. Skin warm. No delirium. Considerable prostration, but she retains sufficient strength to move about, change her position, etc. Abdomen not more tympanitic. Tenderness the same. The inguinal and femoral regions on this day, and previously, were explored for hernial protrusion.

On this morning I introduced a flexible tube into the rectum about 18 inches. It passed thus far with considerable difficulty, even after advancing beyond the sphincter, being detained several times by obstructions which yielded to continued gentle pressure. About a quart of warm water, contningai molasses and castor oil, was injected by means of a forcing syringe attached to the flexible tube. More would have been injected but the tube did not prove sufficiently firm to resist the force of the syringe.

It should have been stated that, on the second day after the attack, i. e., Dec. 1, an enema was given with the common syringe, which was returned with a very little foecal matter; and on the day following, Dec. 2, she had several enemas which came away unaltered. *Castor oil entered into these injections.*

The injection with the flexible tube on the 3d gave no pain, and was shortly returned unchanged. The finger introduced into the rectum passed with facility, and without pain. The uterus was ascertained to be in right position.

Vomiting occasionally occurred during this day, and the patient complained of the taste of castor oil in the matters vomited. It was also stated that oil was observed floating on the fluid vomited. The latter I did not see, but the above was stated without any inquiries being made relative thereto, the patient and friends, too, having no idea of the pathological significancy or importance of the facts. As no oil had been administered *per orem*, it could only have been derived from the enemas.

During the afternoon of the 3d inst. I resolved to try the effect of administering croton oil. She took two pills, each containing a drop of the oil. She retained the first pill a few moments, and severe vomiting then occurred. The second pill was rejected almost instantly, and with severe efforts of vomiting.

Dr. W. visited in consultation on this day, and we again concurred in the propriety of keeping the patient under an anodyne influence, to continue stimulating embrocations over the abdomen, and to cover the abdomen with a thin warm cataplasm.

Dec. 4th. Had passed a tolerably comfortable night. Aspect better. Pulse 100. Skin warm and mellow. Abdomen, as respects tenderness and meteorism, about the same. Resonance over the abdomen tympanitic, except in the *left* iliac region. *No dejection.*

I resolved to try again injection with the flexible tube. Procuring a strong stomach tube, it was introduced without difficulty, about six inches. I could not cause it to advance farther after prolonged efforts, and the exertion of as much force as was deemed prudent. On withdrawing the tube the point gave no evidence of having been in contact with fecal matter. Re-introducing it to the same distance and commencing the injection, in a short time the tube was pushed upward, with facility, about six inches farther. About a quart of warm water, with salt and molasses added, was injected into the intestine, when the patient complained of a sense of distension, and an irresistible desire to evacuate the bowels. Withdrawing the tube the injected fluid came away, containing numerous small flocculi of mucus, tinged with fecal matter. Leaving the patient at this juncture, and returning in about two hours, I found she had passed, in the interim, a large quantity of feces, filling about two thirds of a chamber pot. The feces were of a brown color, of the consistence of mush, not containing any solid portions. The first evacuation was about ten minutes after my departure, and two subsequent evacuations had occurred, all without pain.

The vomiting had much diminished prior to the injection of this morning, and she had been able to keep a little wine and nutriment on the stomach. After the three large evacuations above mentioned, there occurred during the day three small ones. At evening she was comfortable. Pulse about 100, abdomen moderately tympanitic, still tender on pressure, and pressure, as heretofore, (which has not been mentioned,) occasions nausea and efforts to vomit. She is much prostrated, and complains chiefly of a sense of weakness. The treatment now directed was to continue the embrocations, and repeat opiate enemas should there be pain or much restlessness; to give wine, milk, and essence of beef, in small quantities at a time, often repeated.

Dec. 9. This case has progressed favorably since the date of the last record. On the 5th inst. she had a recurrence of severe enteralgic pain, which was relieved by large doses of morphia. She has suffered daily, since, from paroxysms of pain, but on each day less in degree. The pain is referred to the abdomen and back. The spine is very tender on pressure. The abdomen has been moderately tympanitic. The bowels have required enemas, but have been readily moved in that way. She has now some appetite, and gains strength daily. She has had no vomiting since the 4th inst. She is taking Sol. Sulph. Quiniæ, wine, nutritious diet, and pustulation over the spine has been produced by the croton oil.

Up to this date, Jan. 1, 1851, this patient has continued to improve. She is now up and about. She has had no recurrence of vomiting or pain. She is under treatment for Leucorrhea, and is taking chalybeates.

*Remarks.* The interest and novelty of this case seem to the writer to warrant the foregoing detailed report. The history shows it to have been a case of spasmodic, inverted peristaltic action, extending over the whole tract of the intestinal tube. This view of the pathology could not, of course, be deduced from the symptoms prior to the occurrence of the evacuations from the bowels, after an entire obstruction of seven days' duration, on Dec. 4th. Up to that date, after the symptoms assumed a grave character, intus-susceptio, flexion, impacted hardened feces, or some other obstacle to the passage of the intestinal contents, more serious than inverted action, was apprehended. Indeed, this was the view entertained, and the prognosis was accordingly unfavorable. But it is to be observed that while circumstances led to this view of the case, the local and general symptoms were not so intense in degree as they usually are when an irremediable lesion exists. The tenderness over the abdomen was not so extreme, and its neuropathic character was shown by the fact that friction was well borne, and afforded relief. The tympanites was less prominent. The tenderness, moreover, was diffused over the abdomen, and even over the chest, and did not concentrate toward a particular point in the abdomen. It co-existed, too, with highly marked tenderness over the spine, and with paroxysmal pains in the back. The aspect of the patient was not sunken, the muscular strength was not so greatly depressed, and copious perspirations did not occur—these symptoms being, in some measure, characteristic of obstruction dependent on structural lesions. Delirium was not present, as it usually is in the progress of ileus dependent on such lesions. Entertaining, however, notwithstanding these considerations, the supposition that some lesion existed, the propriety of administering cathartics was carefully considered. The natural tendency to prescribe remedies of this class was restrained, under the convictions that if intus-susceptio, or any analagous lesion existed, these remedies would prove ineffectual, that they would interfere with the natural processes upon which the possibility of recovery depended, and that they would tend to exasperate the symptoms, superinducing inflammation, if it did not already exist. The correctness of this view was confirmed by the effects of the single trial of administering croton oil.

It is to be noted in the history of the case, that the symptoms evinced decided improvement prior to the occurrence of dejections from the bowels. On the morning of the day on which the latter took place, the pain and vomiting had diminished, the pulse was less frequent, etc. The occurrence of the dejections, in fact, was doubtless due, in no small degree, to the cessation of the inverted peristaltic motion. The latter stood to the former in the relation of causation, rather than of effect. And, regarding the pathology as involving functional disorder of the muscular tunic of the intestine, not obstruction from a mechanical cause, it is obvious that the measures best calculated to expedite relief, were those which would tend to allay the nervo-muscular irritability of the canal. The injection of a stimulating fluid into the large intestine, however, was doubtless highly serviceable, proving the immediate occasion of the return of the natural peristaltic movements, the inverted action having already ceased. There would perhaps have been an advantage in the treatment of the case, in resorting to this most valuable measure earlier.

The history of this case suggests some inquiries of interest in a physiological, as well as pathological view. The peristaltic movements of the alimentary canal, exclusive of the pharyngeal and anal outlets, are supposed, by physiologists, to be dependent on that portion of the nervous system distinguished as the *sympathetic* or *splanchnic*. They are thought to be independent of the *true spinal* or *reflex* system, the latter presiding over the automatic acts involved in the ingestion and expulsion of the contents of the alimentary canal. Now, do not the circumstances involved in the history of this case render the correctness of this physiological doctrine somewhat questionable? This spasmodic inversion of the peristaltic movements occurred in connection with a well marked affection of the spinal cord—with spinal irritation, as it is usually called. The remote cause, occasioning the attack, seemed to be one acting directly upon the spinal cord, viz., the continued stooping posture, and exertion of the upper extremities involved in quilting—a species of domestic occupation which *tires the backs* even of the healthy and vigorous. The disordered condition of the alimentary canal, moreover, was associated with symptoms due directly to the morbid condition of the cord. Other pathological phenomena, frequently observed in connection with spinal irritation, go to show that the peristaltic movements are not exclusively under the control of the *sympathetic* or *splanchnic* nervous system. Every practitioner who is prepared to appreciate the pathological relations of the cord, has met with cases of obstinate vomiting springing from this source. Tympanites, or meteorism, is also a very common symptom in persons laboring under a morbid condition of the cord, showing defective tone of the contractility of the muscular tunic of the intestines. These facts appear to lead to the conclusion that if the intestinal movements are not dependent on a reflex influence derived from the true spinal system of Marshall Hall, they are, at least, liable to be affected indirectly by morbid influences derived from that source.

---

On consulting the "Principles of Physiology" by Dr. Carpenter, Am. Edition for 1850, since the foregoing article was in type, I find that agreeably to the views entertained by this distinguished Physiologist, the peristaltic movements of the intestinal tube may be influenced, and excited, through the reflex spinal system, although, in his opinion, these movements, normally, are wholly due to the irritability inherent in the muscular tunic of the tube. Dr. Carpenter states that the muscular contractions of the intestine have been observed "to continue long after the tube has been separated from its nervous connections through its whole extent;" and this is supposed to prove that these contractions are independent of any reflex action, either through the spinal cord, or the ganglia of the sympathetic. Experiments on inferior animals, however, as Dr. Carpenter states, show that not only are contractions of the intestinal tube occasioned by irritation of the splanchnic ganglia, but that they follow irritation of the cord, varying their place according to the part of the cord irritated. The author adds, "from these facts it is evident, that the movements of the intestinal tube may be influenced by the spinal cord; and that what is commonly termed the sympathetic nerve, is the channel of that influence, by the fibres which it derives from the spinal system. But it by no means thence follows, that the ordinary peristaltic action of the muscles in question are *dependent* on a stimulus reflected through the spinal cord, rather than on one directly applied to themselves." * * * "The intestinal tube, then, from the stomach to the rectum, is not dependent on the spinal cord for its contractility, but is enabled to propel its contents by its own inherent powers; still we find that here, as in other instances, the nervous centres exert a general control over even the organic functions—doubtless for the purpose of harmonizing them with each other, and with the conditions of the organs of animal life," page 297.

It is obvious that the occurrence of abnormal muscular action of the intestine as the consequence of a morbid condition of the spinal cord, is explicable by application of the physiological views quoted from Dr. Carpenter's work. The reflection, however, arises, that if the peristaltic movements may be thus affected by an influence originating in the spinal cord, it is reasonable to suppose that some influence is derived from the same source in a state of health. It may not, as Dr. Carpenter remarks, follow from the fact of the irritability of the muscular tunic of the intestine being affected by irritating the cord, that the ordinary movements of the former are dependent on the latter; still, it would certainly be a fair presumption that parts thus closely connected in disease, could not be wholly independent of each other in health.

If it be true that the tonic contractility, or tonicity, of the intestinal muscular fibre may be affected by the condition of the cord, as appears to be the case when tympanites occurs as one of the phenomena associated in spinal irritation, this fact would seem to show a more intimate connection between the intestine and the spinal system than does the occurrence of spasmodic contractions as a consequence of causes originating in the latter. The tonicity of the muscles is supposed by Dr. Carpenter, and others, to be a quality inherent in the muscular fibre, not derived from the spinal or other ganglionary centres. The fact, however, just referred to, (if it be a fact,) gives rise to the presumption that tonicity involves an innervation communicated to the muscular structures through its nervous communications. Other pathological facts might be cited in illustration of this opinion. The expression of the face due to the tonicity of the facial muscles, is abolished in paralysis affecting, or involving the portio dura. Here, not only is the power of voluntary motion lost, but the tonic contractility is more or less impaired. Would this be the case if the latter quality were inherent in the muscular fibre, irrespective of its nervous communications? It is granted that this quality may be manifested in muscles after death, and when separated from all their nervous connections. Experiments abundantly establish this to be the case, and, also, that the contractility of muscles may be acted upon, under the same circumstances, so as to produce active movements; may not these facts (which are apparently inconsistent with a relation of dependency of the muscles upon a force of innervation) be explained on the hypothesis that this force, generated within the ganglionary centres of the spinal cord and the sympathetic, and communicated to the muscles, creates, in the latter, properties which continue, for a time, after the supply of the force ceases?

Practitioners who have been accustomed to study cases of spinal irritation, must have observed that a sense of muscular debility, and lassitude, varying in degree at different times, are among the most constant of the host of symptoms appertaining to this affection. Do not these symptoms, referable, as they are, to the condition of the muscular system, denote disordered innervation incident to a morbid condition of the spinal system?

We have thrown out these ideas by way of suggesting inquiries relating to topics interesting both in a physiological and pathological point of view. Much as the functions of the nervous system have been elucidated by modern discoveries and researches, there is still open a wide field for farther developments, which may be expected to advance still more our knowledge of diseases, and their successful management.

